# Inflammatory Bowel Disease and Joint Surgery: A 20-Year Cohort Study of Arthroplasty and Arthritis Risks

**DOI:** 10.7759/cureus.81494

**Published:** 2025-03-31

**Authors:** Nicholas C Bank, Raymond Kim, Bradley J Lauck, Alex Rodriguez-Palacios, R Justin Mistovich

**Affiliations:** 1 Department of Orthopedics, University of North Carolina School of Medicine, Chapel Hill, USA; 2 Department of Gastroenterology, Case Western Reserve University School of Medicine, Cleveland, USA; 3 Department of Orthopedic Surgery, MetroHealth Medical Center, Cleveland, USA

**Keywords:** enteropathic arthritis, extraintestinal manifestations, inflammatory bowel disease, osteoarthritis, total joint arthroplasty

## Abstract

Background

Patients with inflammatory bowel disease (IBD) have a higher risk of adverse outcomes after total hip arthroplasty (THA) and total knee arthroplasty (TKA) compared to the general population. However, existing literature has not fully elucidated the risk of IBD patients to undergo arthroplasty for treatment of arthritis. Therefore, the purpose of this study is to understand the incidence of arthroplasty and osteoarthritis, enteropathic arthritis, or inflammatory polyarthropathies in patients with IBD compared to the general population.

Methods

A retrospective cohort analysis was conducted using the TriNetX research database to identify all patients who experienced the primary outcomes between 2004 and 2024. Two cohorts were stratified by a diagnosis of IBD and propensity score matched (1:1) to mitigate baseline differences in demographics and comorbidities.

Results

After propensity matching, each cohort contained 531,263 patients who were included for analysis. Patients with IBD were significantly less likely to undergo THA (OR 0.853, 95% CI 0.804-0.906) and TKA (OR 0.830, 95% CI 0.788-0.874) and less likely to incur a diagnosis of hip osteoarthritis (OR 0.943, 95% CI 0.922-0.965) or knee osteoarthritis (OR 0.794, 95% CI 0.780-0.808). Conversely, IBD patients were significantly more likely to incur a diagnosis of enteropathic arthropathy (OR 287.9, 95% CI 181.3-457.5) or inflammatory polyarthropathies (OR 1.390, 95% CI 1.365-1.414).

Conclusions

Patients with IBD are less likely to undergo THA and TKA despite the association between IBD and arthritis. These findings underscore the importance of tailored treatment strategies for joint-related complications in IBD patients and highlight the need for further research to optimize surgical outcomes in this population.

## Introduction

Inflammatory bowel disease (IBD) affects nearly seven million people globally and is characterized by chronic inflammation of the intestinal tract by multifactorial etiology [1,2]. Comprised of Crohn’s disease and ulcerative colitis, it is estimated that 5% to 20% of patients with IBD develop peripheral arthritis [3]. Additional rheumatological manifestations of IBD - peripheral arthritis, secondary osteoporosis, secondary hypertrophic osteoarthropathy - are known complications, further leading to joint pain [4]. Although the relationship between IBD and arthritis is not fully understood, it has been hypothesized that inflammatory hindrance of vitamin D and calcium reabsorption, genetic association with HLA-B27, and migration of lymphocytes from gut microbiota to nearby joints may be implicated [3,5-11]. Presently, medical therapy, including NSAIDs, corticosteroids, and immunomodulators, is the mainstay treatment for IBD and its manifestations [2].

Osteoarthritis (OA) is a degenerative joint disease and the most common form of arthritis, affecting an estimated 32.5 million adults in the United States [12]. Characterized by primary or secondary, OA can be a debilitating disease causing pain, stiffness, and inhibited movement. While primary OA develops over time as a function of aging and obesity without trauma or injury, secondary OA is preceded by a joint abnormality accelerating mechanical stress on the joint(s) [13]. While treatment courses may vary based on disease etiology, pharmacological courses may include NSAIDs, glucocorticoid injections, selective serotonin reuptake inhibitors, and opiates, and non-pharmacological courses include weight loss regimens and total joint arthroplasties [13].

According to the CDC, around three million patients in the United States have been diagnosed with IBD, with a higher incidence in those aged 45 and above [14]. Furthermore, the prevalence of IBD is expected to increase over time [15]. As a result, demand for orthopedic surgery for IBD patients experiencing arthritis manifestations may be expected to increase with the aging population [16].

Unfortunately, numerous studies describe a higher risk of adverse outcomes after total hip arthroplasty (THA) or total knee arthroplasty (TKA) procedures in patients with IBD compared to the general population. These risks include postoperative sepsis, thromboembolism, readmission frequency, and fractures [16-20]. Despite the increased prevalence of arthritis and the resultant need for arthroplasty in patients with IBD, as well as a potentially higher complication rate associated with these procedures, existing literature has not fully elucidated the risk of IBD patients to undergo arthroplasty for definitive treatment of their arthritis. Therefore, we have designed a large data, population-based study to understand the incidence of arthroplasty and enteropathic or inflammatory polyarthropathies in patients with IBD compared to the general population. We hypothesized patients with IBD are less likely to undergo traditional arthroplasty due to differences in treatment strategies and potential concerns for higher postoperative risks when compared to the general population.

## Materials and methods

This study utilized the TriNetX database, a global federated health research network comprising 93 participating Healthcare Organizations with more than 131 million patient records with data regarding procedures, diagnoses, medications, and laboratory values [21]. This commercial database was accessed through our institution. This study adhered to the Strengthening the Reporting of Observational Studies in Epidemiology (STROBE) guidelines for consistent reporting of observational data [22]. This study was exempt from IRB review and was a collaborative effort between researchers at UNC Health (Chapel Hill, NC) and MetroHealth/Case Western Reserve University (Cleveland, OH).

Cohort identification

This database was retrospectively queried using International Classification of Diseases, Tenth Revision, Clinical Modification (ICD-10-CM), and Current Procedural Terminology (CPT) coding to identify patients with and without IBD who underwent THA, TKA, or unicompartmental knee arthroplasty (UKA) between 2000 and 2024. Two cohorts were established: cohort 1 (noIBD) included patients without IBD, while cohort 2 (IBD) comprised patients with IBD. Patients were excluded from analysis if they underwent these surgeries prior to having a diagnosis of IBD in their medical records. Primary outcomes of interest were undergoing THA, TKA, or UKA surgery. Secondary outcomes of interest were diagnoses of OA of the hip and/or knee, enteropathic arthritis, and inflammatory polyarthropathy (Table 1).

**Table 1:** Search algorithm used for identification of cohorts and outcomes. IBD, irritable bowel disease; ICD, International Classification of Disease; CPT, Current Procedural Terminology

IBD cohort	Procedure or diagnosis
ICD-10: K50	Crohn’s disease
ICD-10: K51	Ulcerative colitis
Outcomes	
CPT: 27130	Arthroplasty, acetabular and proximal femoral prosthetic replacement (total hip arthroplasty), with or without autograft or allograft
CPT: 27447	Arthroplasty, knee, condyle, and plateau; medial AND lateral compartments with or without patella resurfacing (total knee arthroplasty)
CPT: 27446	Arthroplasty, knee, condyle, and plateau; medial OR lateral compartment
ICD-10: M16	Osteoarthritis of hip
ICD-10: M17	Osteoarthritis of knee
ICD-10: M07	Enteropathic arthropathies
ICD-10: M05-M14	Inflammatory polyarthropathies

Statistical analysis

To minimize potential confounding, cohorts were matched by 1:1 propensity scoring on the basis of age, sex, obesity, and race. The ICD-10-CM and CPT codes used for cohort identification, matching, and outcomes are detailed in Table 1. Outcomes were compared using odds ratios (OR) and 95% confidence intervals (95% CI). Chi-square tests were used to determine differences in categorical comorbidity variables, and Student’s t-tests were used to analyze differences in continuous comorbidity variables, as indicated. A p-value of <0.05 was used to determine statistical significance. All statistical analysis was performed using the TriNetX database analysis software.

## Results

A total of 551,055 patients with IBD and 15,382,067 patients without inflammatory bowel disease (noIBD) were identified based on inclusion and exclusion criteria. After propensity score matching, both cohorts contained 531,263 patients each (Table 2).

**Table 2:** Cohort counts before and after propensity score matching. IBD, irritable bowel disease; noIBD, without irritable bowel disease

Cohort	Before matching	After matching
IBD	551,055	531,263
noIBD	15,382,067	531,263

Cohort demographics

Before Propensity Score Matching

Patients in the IBD cohort were significantly older (p<0.001), more likely to be female (p<0.001), and more likely to be White (p<0.001). Patients in the noIBD cohort were more likely to be Black or African American (p<0.001), Native Hawaiian or Other Pacific Islander (p<0.001), and Hispanic or Latino (p<0.001). Obesity was significantly more common in the IBD group before matching (p<0.001). The baseline characteristics of patients with and without IBD prior to matching are summarized in Table 3.

**Table 3:** Cohort demographics and comorbidities before 1:1 propensity matching of patients with IBD (1) vs. without IBD (2). Chi-square tests were used to determine differences in categorical comorbidity variables, while Student’s t-tests were used to analyze differences in continuous comorbidity variables. IBD, irritable bowel disease

Cohort	Characteristic	Mean ± SD	Patients	% of Cohort	*P*	Std diff.
1	Age at index	46.8 ± 20.2	531,263	100%	<0.001	0.511
2		35.1 ± 25.1	15,111,794	100%		
1	Female		276,212	52.0%	<0.001	0.012
2			7,763,623	51.4%		
1	Black or African American		45,553	8.6%	<0.001	0.163
2			2,068,901	13.7%		
1	Male		227,200	42.8%	<0.001	0.005
2			6,503,163	43.0%		
1	White		377,431	71.0%	<0.001	0.256
2			8,905,117	58.9%		
1	American Indian or Alaska Native		1,355	0.3%	<0.001	0.013
2			49,196	0.3%		
1	Unknown race		76,068	14.3%	<0.001	0.103
2			2,735,779	18.1%		
1	Native Hawaiian or Other Pacific Islander		1,182	0.2%	<0.001	0.010
2			41,033	0.3%		
1	Unknown ethnicity		117,963	22.2%	<0.001	0.039
2			3,601,295	23.8%		
1	Not Hispanic or Latino		386,664	72.8%	<0.001	0.180
2			9,741,498	64.5%		
1	Hispanic or Latino		26,636	5.0%	<0.001	0.244
2			1,769,001	11.7%		
1	Other race		17,685	3.3%	<0.001	0.074
2			722,515	4.8%		
1	Asian		11,989	2.3%	<0.001	0.095
2			589,253	3.9%		
1	Overweight and obesity		41,907	7.9%	<0.001	0.041
2			1,029,934	6.8%		

After Propensity Score Matching

**Figure 1:**
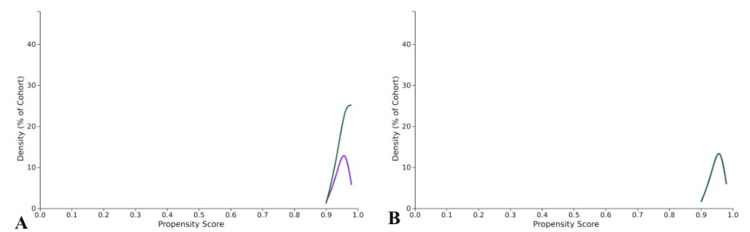
Propensity score density plot before and after 1:1 propensity matching. Panel A displays the propensity score density plot before cohort matching. The green line represents the cohort without IBD, while the purple line represents the cohort with IBD. Panel B displays the propensity score density plot after matching. The green line represents both cohorts overlapped, indicating a well-matched cohort.

After matching, the cohorts did not differ in age, sex, or prevalence of obesity. The IBD cohort was more likely to be Native Hawaiian or Other Pacific Islander (0.3% vs. 0.2%, p=0.005), while the noIBD cohort was more likely to be Hispanic or Latino (5.0% vs. 8.6%, p<0.001). The baseline characteristics of patients with and without IBD after matching are summarized in Table 4. Propensity score density plots indicate two well-balanced groups were obtained (Figure 1).

**Table 4:** Cohort demographics and comorbidities after 1:1 propensity matching of patients with IBD (1) vs. without IBD (2). Chi-square tests were used to determine differences in categorical comorbidity variables, while Student’s t-tests were used to analyze differences in continuous comorbidity variables. IBD, irritable bowel disease

Cohort	Characteristic	Mean ± SD	Patients	% of Cohort	*P*	Std diff.
1	Age at index	46.8 ± 20.2	531,263	100%	0.999	<0.001
2		46.8 ± 20.2	531,263	100%		
1	Female		276,212	52.0%	0.997	<0.001
2			276,214	52.0%		
1	Black or African American		45,553	8.6%	0.992	<0.001
2			45,550	8.6%		
1	Male		227,200	42.8%	0.995	<0.001
2			227,203	42.8%		
1	White		377,431	71.0%	0.998	<0.001
2			377,430	71.0%		
1	American Indian or Alaska Native		1,355	0.3%	0.001	0.006
2			1,192	0.2%		
1	Unknown race		76,068	14.3%	<0.001	0.010
2			74,179	14.0%		
1	Native Hawaiian or Other Pacific Islander		1,182	0.2%	0.005	0.005
2			1,049	0.2%		
1	Unknown ethnicity		117,963	22.2%	<0.001	0.012
2			120,594	22.7%		
1	Not Hispanic or Latino		386,664	72.8%	<0.001	0.089
2			365,138	68.7%		
1	Hispanic or Latino		26,636	5.0%	<0.001	0.142
2			45,531	8.6%		
1	Other race		17,685	3.3%	<0.001	0.017
2			16,129	3.0%		
1	Asian		11,989	2.3%	<0.001	0.044
2			15,734	3.0%		
1	Overweight and obesity		41,907	7.9%	0.994	<0.001
2			41,905	7.9%		

Primary outcomes

The IBD group was less likely to undergo THA (OR 0.853, 95% CI 0.804-0.906), TKA (OR 0.830, 95% CI 0.788-0.874), and UKA (OR 0.755, 95% CI 0.597-0.954) during the observed period. The IBD cohort was also significantly less likely to incur a diagnosis of hip OA (OR 0.943, 95% CI 0.922-0.965) and knee OA (OR 0.794, 95% CI 0.780-0.808).

Conversely, the IBD cohort was more likely to incur a diagnosis of enteropathic arthropathy (OR 287.98, 95% CI 181.286-457.457) or inflammatory polyarthropathy (OR 1.390, 95% CI 1.365-1.414). While there is a small absolute difference in the percentage risks of the cohorts for outcomes, due to the larger population size, the differences in risks and odds still correlate as statistically significant differences. These findings are presented in Table 5.

**Table 5:** Odds ratios and risk differences for primary outcomes.

Outcome	Odds ratio (95% CI)	Risk difference (95% CI)
Total hip arthroplasty	0.853 (0.804, 0.906)	-0.001 (-0.001, -0.000)
Total knee arthroplasty	0.830 (0.788, 0.874)	-0.001 (-0.001, -0.001)
Unicompartmental knee arthroplasty	0.755 (0.597, 0.954)	-0.000 (-0.000, -0.000)
Hip osteoarthritis	0.943 (0.922, 0.965)	-0.002 (-0.002, -0.001)
Knee osteoarthritis	0.794 (0.780, 0.808)	-0.011 (-0.012, -0.010)
Enteropathic arthropathy	287.9 (181.3, 457.5)	0.010 (0.009, 0.010)
Inflammatory polyarthropathy	1.390 (1.365, 1.414)	0.017 (0.016, 0.017)

## Discussion

IBD affects approximately three million people in the United States, has significant associations with arthritis, and is expected to increase in prevalence in the population [16]. Although prior literature has alluded to poorer outcomes of total joint procedures for patients with IBD, much less is known regarding overall rates of OA and risk of TJA in this unique population [16-18,20]. Therefore, this study utilized a large population database to elucidate this relationship.

The primary finding of our study shows that patients with IBD are actually less likely to undergo THA, TKA, or UKA than those without IBD. Furthermore, IBD patients are less likely to be diagnosed with OA of the hip and knee compared to those without IBD. Conversely, IBD patients were much more likely to be diagnosed with enteropathic arthritis and inflammatory polyarthropathy. This discrepancy likely reflects the differences in disease pathology giving rise to the hip and/or knee arthritis in these patients. While concurrent OA may be an underdiagnosed contributing factor, it is clinically challenging to quantify the degree of joint pain caused by any given etiology.

Additionally, our results show that total joint arthroplasties are less common among the IBD cohort. The emergence of different biological therapies has led to a robust treatment course indicated for gastroenteric pathologies [3]. The initial management of peripheral enteropathic arthritis begins with NSAIDs and local glucocorticoids, with more progressive symptoms indicating antirheumatic, TNF inhibitors, and interleukin inhibitors [3,23]. Since medical management of IBD helps to resolve symptoms of concurrent arthropathy, anti-inflammatory/immunomodulating medication courses are likely prioritized over considerations for total hip and knee arthroplasty procedures. In a recent literature review of IBD and arthroplasty, Arvikar and Fisher found peripheral arthritis in 2.8% to 31% of patients with IBD [5], which may be an underestimation due to the transient nature of arthritis and impact of arthritis-related steroids administered for IBD flares. This further supports the findings of Ehrenpreis and Zhou’s study [24], which found that hip arthroplasty and knee arthroplasty are less prevalent in hospitalized patients with IBD, as patients diagnosed with IBD underwent more intensive screenings and treatment for OA, limiting progression to need for THAs.

Moreover, many studies have emphasized poorer outcomes of THA and TKA procedures for patients with IBD, citing increased risk of infections, revisions, and readmissions [16-20,24]. Post-THA survivorship was found to be lower among IBD patients as well. Kapadia et al.’s 10-year retrospective study found a higher mortality rate among the IBD cohort, as well as higher rates of septic complications [17]. Additionally, Magruder et al.’s assessment of post-operative outcomes for IBD patients found significantly higher rates of ED re-admissions for post-THA complications, which contributed to increased economic burden [19]. Given this understanding, clinicians and surgeons are likely more reserved in recommending surgical intervention for these patients.

This study was not without limitations. Inherently, large population databases rely on the accuracy and precision of coding and billing of providers and healthcare systems. Errors here may manifest as inaccurate cohort samples and outcome prevalences - either over- or underestimated. To mitigate such inaccuracies, TriNetX employs a comprehensive data quality maximizing methodology including a growing data quality metric library, data harmonization, site benchmarking, and semantic marking [25].

## Conclusions

Overall, this study demonstrates significant relationships between patients with IBD, types of arthritis, and risk of subsequent arthroplasty. These findings underscore the need to promote patient awareness of the risk of total joint procedures in the setting of their IBD and to encourage physicians to critically evaluate all available data (radiographic, laboratory, clinical) to best ascertain the etiology of a patient’s hip or knee pain. Moreover, this study highlights the need for more research on the pathophysiology of enteropathic arthritis and innovations in safe and effective procedure modalities to increase surgical outcomes in these patients.
